# miR‐26a‐5p alleviates CFA‐induced chronic inflammatory hyperalgesia through Wnt5a/CaMKII/NFAT signaling in mice

**DOI:** 10.1111/cns.14099

**Published:** 2023-02-08

**Authors:** Yitian Lu, Maozhu Liu, Xiangna Guo, Peng Wang, Fanning Zeng, Haitao Wang, Jing Tang, Zaisheng Qin, Tao Tao

**Affiliations:** ^1^ Department of Anesthesiology, Nanfang hospital Southern Medical University Guangzhou China; ^2^ Department of Anesthesiology Central People's Hospital of Zhanjiang Zhanjiang China; ^3^ Department of pharmacy West China Hospital, Sichuan University Chengdu China; ^4^ School of Pharmaceutical Sciences Southern Medical University Guangzhou China; ^5^ Department of Anesthesiology Affiliated Hospital of Guangdong Medical University Zhanjiang China

**Keywords:** inflammatory pain, miR‐26a‐5p, neuroinflammation, Wnt5a

## Abstract

**Background:**

Inflammation often leads to the occurrence of chronic pain, and many miRNAs have been shown to play a key role in the development of inflammatory pain. However, whether miR‐26a‐5p relieves pain induced by inflammation and its possible mechanism are still unclear.

**Methods:**

The complete Freund's adjuvant (CFA)‐induced inflammatory pain mouse model was employed. Intrathecal or subcutaneous injection of miR‐26a‐5p agomir was performed after modeling to study its antinociceptive effect and the comparison of different administration methods. Bioinformatics analysis of miRNAs was performed to study the downstream mechanisms of miR‐26a‐5p. HE staining, RT‐qPCR, Western blotting, and immunofluorescence were used for further validation.

**Results:**

A single intrathecal and subcutaneous injection of miR‐26a‐5p both reversed mechanical hypersensitivity and thermal latency in the left hind paw of mice with CFA‐induced inflammatory pain. HE staining and immunofluorescence studies found that both administrations of miR‐26a‐5p alleviated inflammation in the periphery and spinal cord. Bioinformatics analysis and dual‐luciferase reporter gene analysis identified Wnt5a as a direct downstream target gene of miR‐26a‐5p. Wnt5a was mainly expressed in neurons and microglia in the spinal cord of mice with inflammatory pain. Intrathecal injection of miR‐26a‐5p could significantly reduce the expression level of Wnt5a and inhibit the downstream molecules of noncanonical Wnt signaling Camk2/NFAT, inhibiting the release of spinal cord inflammatory factors and alleviating the activation of microglia. In addition, miR‐26a‐5p could also inhibit lipopolysaccharide (LPS)‐stimulated BV2 cell inflammation in vitro through a noncanonical Wnt signaling pathway.

**Conclusions:**

miR‐26a‐5p is a promising therapy for CFA‐induced inflammatory pain. Both intrathecal and subcutaneous injections provide relief for inflammatory pain. miR‐26a‐5p regulated noncanonical Wnt signaling to be involved in analgesia partly through antineuroinflammation, suggesting a pain‐alleviating effect via noncanonical Wnt signaling pathway in the CFA‐induced inflammatory pain model in vivo.

## Graphical abstract

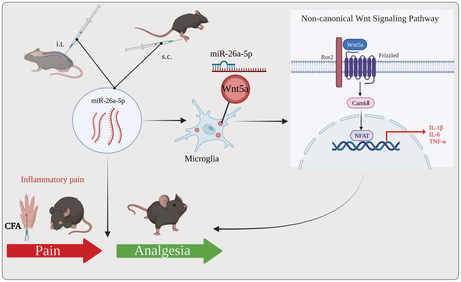

## 1 BACKGROUND

Inflammation is a vital physiological response to noxious stimuli (e.g., trauma, infection, and surgery), which facilitates to repair the impairment and restore the homeostasis.^1^, ^2^, ^3^ While the inflammation maintains 6 weeks or longer, for example, dermatitis, arthritis, and colitis, inflammation would exhibit its evil nature resulted in persistent damage. Inflammatory factors, for example, IL‐1β, TNF‐α, and prostaglandin, produced during chronic inflammation could sensitize peripheral nerve resulted in suffering chronic pain.^4^ Chronic inflammatory pain has become one of the major public health problems worldwide, seriously affecting the quality of patients' life and causing a huge economic burden.^5^, ^6^ A recent population‐based survey in Europe found that 25%–35% of adults report chronic inflammatory pain.^5^, ^7^, ^8^ Another study showed that 79% of patients with chronic inflammatory pain still had some degree of chronic pain 4 years later.^9^ Therefore, the clinical management of chronic inflammatory pain remains a great challenge.^10^ The drugs currently used to treat chronic inflammatory pain mainly include non‐steroidal anti‐inflammatory drugs (NSAIDs), opioids, and other adjuvant drugs, but most of them have many side effects and the treatment efficacy is not satisfied.^11^ There is an urgent need to redefine and improve the pain management strategy (e.g., cellular therapy and nuclear acid medicine) to relief inflammatory pain.

Inflammatory pain is closely related with immune response and is normally mimicked in animals through exerting pro‐inflammatory stimuli, such as tissue incision, complete Freund's adjuvant (CFA), and lipopolysaccharides (LPS).^4^ During the acute phase, pain is usually proportional to the inflammation. While there is the development into the chronic phase, the persistent inflammation triggers cytokines and irritant substances to regulate the peripheral and central mechanisms of pain through the neuroimmune mechanism, which would ultimately result in hyperalgesia, allodynia, and idiopathic pain.^12^ During inflammation, immune cells release mediators that act on the peripheral nerve terminals of nociceptive neurons.^13^ Action potentials are transmitted through the dorsal root ganglion (DRG) to the spinal cord, where they are eventually relayed to the brain and processed as pain. In the dorsal horn of the spinal cord, neuroimmune interactions contribute to the central mechanisms of pain.^14^ Neuroinflammation act as a key mechanism in the pathogenesis of chronic inflammatory pain,^15^ and in inflammatory pain states, neuroinflammation involves activation of microglia, release of pro‐inflammatory mediators, and overexpression of pain‐related signaling molecules. Primary nociceptive afferent nerves in the DRG release glutamate, ATP, and chemokines from their central terminals. T cells, microglia, and astrocytes also produce pro‐inflammatory cytokines and growth factors that act presynaptically and postsynaptically to stimulate nerve endings to increase nerve conduction and mediate central pain sensitization. Immune cells release mediators directly sensed by nociceptor terminals to modulate neuronal excitation and pain transduction. IL‐1β, tumor necrosis factor (TNF), nerve growth factor (NGF), and prostaglandin E2 (PGE2), etc. bind to cognate receptors expressed at nociceptor terminals to mediate neuronal firing.^12^ The overall result of these immune‐mediated pathways in nociceptors is a lowering of the threshold for responses to mechanical or thermal stimuli, leading to increased pain sensitivity. Therefore, targeting these signaling molecules and/or cells may be a potential therapeutic strategy for chronic inflammatory pain.^10^

miRNAs are short and endogenous noncoding RNAs which regulate mRNA expression through inhibiting mRNA expression and/or reducing mRNA stability.^16^, ^17^ In the past decades, miRNAs not only have been developed as biomarkers for diagnosis, prognosis prediction, but also have been considered as a potential and attractive target for developing new therapies of different diseases, including cancers, cardiovascular diseases, and diabetes.^18^, ^19^ Many previous studies have shown that miRNAs have different roles during the pathophysiological process of inflammatory pain.^20^, ^21^ miRNA‐107 promotes inflammatory pain by downregulating GLT‐1 expression in the dorsal horn of the rat spinal cord.^22^ miRNA‐22 upregulates Mtf1 in dorsal horn neurons to induce and maintain inflammatory pain,^23^ while elevated miR‐451 alleviates inflammatory pain by targeting TLR4 to suppress microglial activation‐induced inflammatory responses,^24^ miR‐485‐5p alleviates inflammatory pain by inhibiting the transcription of ASIC1 in rat dorsal root ganglia,^25^ what's more, microRNA‐141‐3p alleviates chronic inflammatory pain by downregulating downstream target gene HMGB1.^26^ In our previous study, we found that miR‐26a‐5p in small extracellular vesicles (sEVs) derived from human placental mesenchymal stem cells (hPMSCs) could alleviate peripheral nerve injury‐induced neuropathic pain by decreasing spinal neuroinflammation via Wnt5a, a noncanonical Wnt signaling pathway.^27^ Additionally, miR‐26a‐5p also exhibits its antiinflammatory potential in other disease models. miR‐26a‐5p/IL‐6 axis alleviates sepsis‐induced acute kidney injury by inhibiting renal inflammation.^28^ Overexpression of miR‐26a‐5p could alleviate acute lung injury by targeting TLR4 to reduce cellular inflammation.^29^ Another previously study also reported that miR‐26a‐5p alleviates neuropathic pain in a rat model of CCI through decreasing the release of the inflammatory factors IL‐1β, IL‐6, and TNF‐α by targeting MAPK6.^30^ However, whether miR‐26a‐5p could alleviate inflammatory pain through its antiinflammatory effect and its potential target molecules is still need to be further explored.

Therefore, in the current study, we examined the analgesic effect of miR‐26a‐5p in CFA‐induced inflammatory pain mice model. In addition, we compared the analgesic effects of two different miR‐26a‐5p administration methods (intrathecal and subcutaneous). Finally, we demonstrate that miR‐26a‐5p and its target gene Wnt5a play an important role in allodynia by reducing microglial activation in the spinal dorsal horn and suppressing inflammation through noncanonical Wnt signaling pathway.

## 2 MATERIALS AND METHODS

### 2.1 Animal and pain models

Male C57/BL6 mice (8 weeks old, 22 ± 2 g) were obtained from the central animal facility of Southern Medical University (Guangzhou, China). The animals were housed under standard conditions of light and dark cycles (12 h:12 h, temperature 25°C) with free access to food and water. They were allowed to acclimate to these conditions for at least 3 days before all experiments. Inflammatory pain was established in mice by subcutaneous injection of CFA (20 μL, Sigma, St. Louis, MO) to the left plantar after mice were anesthetized with sevoflurane (2%–5%). For mice in sham group, 20 μL of normal saline was subcutaneously injected into the left paw. Twenty‐four hours after intrathecal or subcutaneous injection, mouse spinal cord or hind paw subcutaneous tissue were taken for experiment. All the animal studies were carried out according to the approved protocols and guidelines of the Institutional Animal Ethical Care Committee of Southern Medical University Experimental Animal Centre and the International Association for the Study of Pain.^31^

### 2.2 Cell culture and treatment

BV2 cell line was obtained from Gaining Biological Technology Co., Ltd (Shanghai, China). Immortalized mouse BV2 microglial cell lines were cultured in DMEM medium with 10% FBS and 1% penicillin/streptomycin at 37°C in a humidified atmosphere containing 95% air and 5% CO_2_, and the medium was changed daily. The cells were digested with 0.25% trypsin when they reached 70% confluence and subcultured for further passages. BV2 cells were transfected with miR‐26a‐5p agomir using lipofectamine 2000 (Invitrogen) in accordance with the manufacturer's recommendations for 48 h and treated with or without LPS (1 μg/mL) and Foxy5 (1 μg/mL) for 24 h.

### 2.3 Drugs and reagents

CFA (#F5881) was purchased from Sigma‐Aldrich. Foxy5(#S6961) was purchased from Selleck. Foxy5 was dissolved in DMSO at a concentration of 1 mg/mL. mmu‐miR‐26a‐5p agomir, agomir‐NC and miR‐26a‐5p mimic, and mimic‐NC were all purchased from GenePharma Co. Ltd. (Shanghai, China). The mmu‐miR‐26a‐5p agomir dissolved concentration was 200 nmoL/mL in DEPC‐treated Water.

### 2.4 Intrathecal injections

For intrathecal injection, we use the method described previously.^32^ Mice were anesthetized with isoflurane. The thumb and middle finger of the left hand firmly hold the paralumbar region of the iliac crest, and the index finger is placed on the tip of the spinous process of the sixth lumbar vertebra (L6), the highest point of the vertebral body. All intrathecal injections were delivered in a total volume of 10 μL. The needle is inserted into the fifth intervertebral space (L5–L6), resulting in a sudden lateral movement of the tail. The needle is held in place for 10 s and then slowly withdrawn to avoid spillage.

### 2.5 Behavioral test

All mice were acclimated to the test environment for 30 min before testing for baseline nociceptive thresholds. The mechanic threshold of mice was measured by using the von Frey monofilament (Semmes Weinstein) “up‐down” method.^33^ Mice were placed in glass boxes on grid iron racks (9 × 25 × 25 cm). At the beginning of the test, von Frey filament was applied to the plantar surface of the hind paw. Bend and hold the filament for 3 s. The researchers were blinded to the treatment group during behavioral testing. The threshold force required to retract the hind paw each time was measured and averaged (with a minimum interval of 20 min between measurements). For the Hargreaves test, infrared heat was applied to the plantar surface of the hind paw using a Hargreaves device (Ugo Basile) and the latency to exit the paw (thermal latency) was measured, using 20 s as the cutoff latency. Thermal tests were repeated three times at 20‐min intervals, and the mean value was calculated.

### 2.6 Bioinformatic analysis of microRNA target genes

The following four databases were used to predict the putative target genes of miR‐26a‐5p: microT,^34^ miRanda, PicTar,^35^ and TargetScan.^36^ Venn diagrams were used to screen the intersection of predicted target genes from four databases. DAVID Bioinformatics Resources 6.82 was used to analyze bioinformatics data for Gene Ontology enrichment of common target genes predicted by the four databases.^37^ Statistical analysis and visualization were carried out in R version 3.6.3. R packages involved cluster Profiler package (for enrichment analysis and visualization) and ggplot2 package (for visualization).

### 2.7 Dual‐luciferase reporter assay

The luciferase assay was performed with a dual‐luciferase reporter system (Promega). Briefly, wild‐type (WT) or mutant (MUT) Wnt5a 3′‐UTR reporter constructs were co‐transfected into BV2 cells with the miR‐26a‐5p‐mimic or negative control (NC), using lipofectamine 2000 (Invitrogen, USA), followed by the analysis of luciferase activities, in which Renilla was applied as a normalized control. The specific system of transfection is as follows: Solution A: Dilute pmirGLO‐Wnt5a‐3′UTR/mut 0.4 μg with serum‐free medium Opti‐MEM, final volume 50 μL, Solution B: Dilute 0.4 μL Lipofectamine 2000 Reagent with serum‐free medium Opti‐MEM, final volume 50 μL. When Renilla luciferase was used as the internal reference, the RLU value obtained by the firefly luciferase assay was divided by the RLU value obtained by the Renilla luciferase assay. Based on the ratios obtained, the activation levels of target reporter genes were compared between different samples.

### 2.8 RNA Extraction and quantitative real‐time polymerase chain reaction (qRT‐PCR)

Total RNA isolated from BV2 cells, spinal cord tissue, and subcutaneous tissue of the hind paw using TRIzol reagent (Thermo Fisher Scientific) was used for the subsequent qPCR verification. In brief, total RNA samples were used for cDNA library preparation using a PrimeScript RT reagent Kit (TaKaRa). mRNA expression was determined by quantitative real‐time polymerase chain reaction (qRT‐PCR) using a SYBR Premix Ex TaqTM II (Tli RNaseH Plus) kit (TaKaRa). qRT‐PCR was performed on the ABI QuantStudio six flex (Applied Biosystems, United States). The PCR reaction was performed as follows: cycling conditions began with an initial DNA denaturation step at 95°C for 20 s, followed by 40 cycles at 94°C for 15 s, 56°C for 30 s, and 72°C for 25 s. Glyceraldehyde 3‐phosphate dehydrogenase (GAPDH) was the normalization for the quantification of gene expression, using the ΔΔCt method. The primers for mouse genes were synthesized by RiboBio (GuangZhou, China), and sequences can be found in Table 1.

**TABLE 1:** Sequence of primers.

Gene	Primer sequences (5′–3′)	
	Forward	Reverse
GAPDH	TTGCTGTTGAAGTCGCAGGAG	TGTGTCCGTCGTGGATCTGA
Wnt5a	CAACTGGCAGGACTTTCTCAA	CATCTCCGATGCCGGAACT
CaMKII	TATCCGCATCACTCAGTACCTG	GAAGTGGACGATCTGCCATTT
NFAT	CAGTGTGACCGAAGATACCTGG	TCGAGACTTGATAGGGACCCC
IL‐1β	TGTAATGAAAGACGGCACACC	TCTTCTTTGGGTATTGCTTGG
TNF‐α	AATGGCCTCCCTCTCATCAGTTCT	TGAGATAGCAAATCGGCTGACGGT
IL‐6	AATTAAGCCTCCGACTTGTGAAG	CTTCCATCCAGTTGCCTTCTTG
IL‐10	GCTCTTACTGACTGGCATGAG	CGCAGCTCTAGGAGCATGTG
TGF‐β	CTCCCGTGGCTTCTAGTGC	GCCTTAGTTTGGACAGGATCTG

### 2.9 Western blotting

BV2 cells and mouse spinal cord L4‐5 segment tissues were digested in RIPA extraction buffer (Beyotime, China). Protein samples were separated by 10% SDS‐PAGE and transferred onto polyvinylidene difluoride (PVDF) membranes (Millipore, United States) in tank transfer system (Bio‐Rad, United States). Membranes were blocked with 5% nonfat milk in buffer containing 0.1% Tween‐20 (TBST) for 1 h, washed three times in TBST, and incubated overnight at 4°C with primary antibodies including rabbit anti‐Wnt5a (1:1000 dilution; Proteintech; USA; 55,184‐1‐AP), rabbit anti‐CaMKII (1:1000 dilution; Proteintech; USA; 13,730‐1‐AP), rabbit anti‐NFAT (1:1000 dilution; Proteintech; US; 22,023‐1‐AP), rabbit anti‐GAPDH (1:10000 dilution; Proteintech; USA; 10,494‐1‐AP), rabbit anti‐iNOS (1:1000 dilution; Proteintech; US; 18,985‐1‐AP), and rabbit anti‐CD206(1:1000 dilution; Proteintech; US; 18,704‐1‐AP). After incubation with the HRP‐conjugated goat anti‐rabbit IgG secondary antibody (1:10000 dilution, Da‐UN, China), immunoreactive bands were detected by enhanced chemiluminescence (Millipore, United States). The protein bands were quantitatively analyzed using ImageJ software 1.8.0 (National Institutes of Health, Bethesda, MA, USA).

### 2.10 ELISA for the determination of cytokine levels

A cell suspension was prepared by inoculating BV2 microglial cells in the logarithmic growth phase into a 6‐well culture plate. After BV2 cells were transfected with miR‐26a‐5p agomir for 48 h and treated with or without LPS (1 μg/mL) and Foxy5 (1 μg/mL) for 24 h, the supernatant was collected. Similarly, spinal cord L4‐5 of mice were lysed after different treatments, and cytokine levels were determined. The concentrations of IL‐1β, TND‐α, TGF‐β, and IL‐10 levels were measured by ELISA according to the manufacturer's instructions (Proteintech). Optical density (OD) was measured at 450 nm using a microplate reader (Thermo Scientific).

### 2.11 H&E staining

Twenty‐four hours after intrathecal or subcutaneous injection, mouse hind paw subcutaneous tissues were taken for experiment. Mice were anesthetized with pentobarbital (0.3%) and perfused transcardially with 37°C saline followed by 4% paraformaldehyde in 0.1 M PBS (pH, 7.4; 4°C). The tissue was then immediately removed and fixed in 10% buffered formalin for 48 h in preparation for routine paraffin histology. Paraffin‐embedded 5 μm‐thick sections from different groups were stained with hematoxylin and eosin (H&E).

### 2.12 Immunofluorescence staining

Subcutaneous tissue of the hind paw and ipsilateral L4‐5 spinal cord samples were removed and cut into 20‐μm frozen cryosections using a microtome. Tissue sections were fixed for 10 min in 4% paraformaldehyde (Solarbio, China) at room temperature, then permeabilized and blocked with 0.5% TritonX‐100 (Sigma‐Aldrich) and 3% bovine serum albumin (BSA, Solarbio, China) at room temperature for 1 h. Cell climbing slices were placed in a 6‐well plate. BV2 cells were cultured and treated as indicated and fixed with 4% paraformaldehyde for 30 min at room temperature followed by permeabilization using 0.3% Triton X‐100 for 15 min. The cells were blocked with 3% bovine serum albumin in PBST for 1 h. Next, BV2 cell climbing slices, the hind paw, and spinal cord tissue sections were incubated with diluted primary antibodies against CD206 (Proteintech 18,704‐1‐AP), Iba1 (Wako 559–24,761), GFAP (Proteintech 16,825‐1‐AP), NeuN (GeneTex GTX132974), iNOS (Proteintech 18,985‐1‐AP), and Wnt5a (Santa Cruz sc‐365,370). Sections were then incubated with appropriate secondary antibodies (1:500, Alexa Fluor 488‐labeled goat anti‐rabbit, mouse IgG, Jackson Immuno Research, West Grove, PA; 1:200, CoraLite594‐conjugated Goat Anti‐Mouse IgG (H + L), Proteintech, SA00013‐3) for 1 h at room temperature. Finally, the slides were mounted with antiquenching DAPI (49,6‐diamino‐2‐phenylindole) fluorescent mounting medium. Images were acquired with upright manual fluorescence microscope (Zeiss, ImagerD2, Germany), then processed with Adobe Photoshop 8.0 software (Adobe Systems, Mountain View, CA). When counting the number of positive cells, cell crawls were taken in the 3,6,9,12 o'clock direction and central field of view for analysis. A total of three independent samples were taken, and the observer was blinded to the grouping. Semi‐quantitative analysis of fluorescence images of iNOS and IBA1 was performed with ImageJ software 1.8.0 (National Institutes of Health, Bethesda, MA, USA).

### 2.13 Statistical analysis

Statistical analyses were performed using SPSS 22.0 Statistics (IBM SPSS Statistics for Version 22.0, IBM Corp, North Castle). All data are expressed as mean ± SEM. For data obtained via behavioral test data, two‐way ANOVA with repeated measures followed by Tukey's post hoc test was used to analyze the differences between different groups. For data obtained via qRT‐PCR, Western blotting, Elisa, and immunofluorescence staining, one‐way ANOVA followed by Tukey's post hoc test was used for multiple group comparisons. Shapiro–Wilk normality test indicated that the data have normal distribution; therefore, comparisons were done using parametric tests. Differences were statistically significant when *p* value was <0.05.

## 3 RESULTS

### 3.1 miR‐26a‐5p alleviates complete Freund's adjuvant‐induced inflammatory pain and suppresses inflammation by a single intrathecal or subcutaneous injection

**FIGURE 1:**
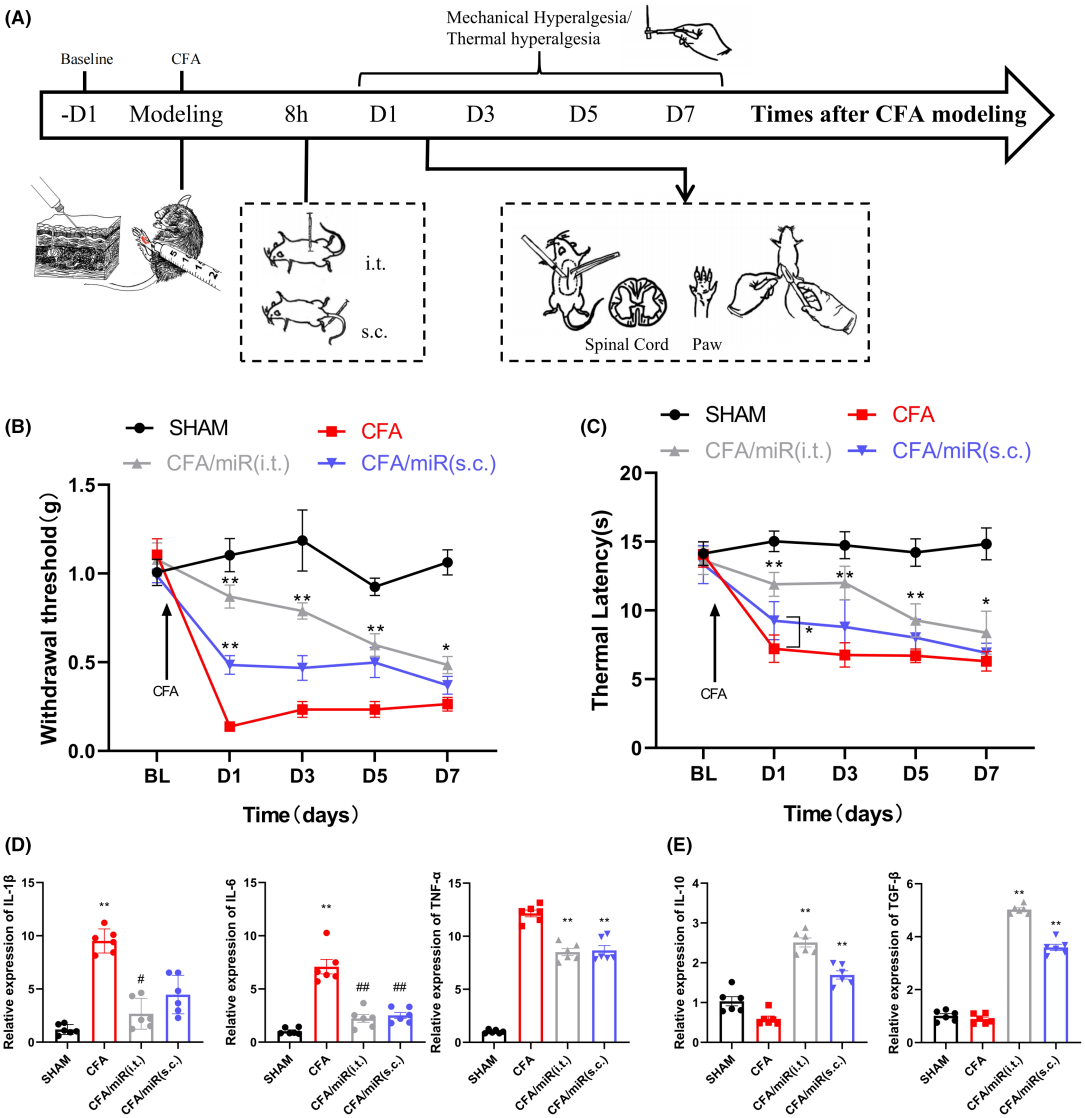
miR‐26a‐5p alleviates complete Freund's adjuvant‐induced inflammatory pain and suppresses inflammation by a single intrathecal or subcutaneous injection. (A) Experimental protocol for establishing complete Freund's adjuvant‐induced inflammatory pain mouse model, tissue collection, and pain behavioral test. (B) 50% paw withdraw threshold (PWT) of left hind paw of mice in different treatment groups mice. At day 1, the paw withdrawal thresholds (WTs) of CFA/miR(s.c.) group were significantly higher than those of Sham group after single injection. At day 1, 3, 5 and 7, the WTs of CFA/miR(i.t.) group were significantly higher than those of Sham group after single injection. (C) Thermal latency(s) of left hind paw of mice in different treatment groups. At day 1, the thermal latency(s) of CFA/miR(s.c.) group was significantly higher than those of Sham group after single injection. At day 1, 3, 5 and 7, the thermal latency(s) of CFA/miR(i.t.) group was significantly higher than those of Sham group after single injection (*p < 0.05, **p < 0.01 compared with CFA/miR(i.t.) and CFA/miR(s.c.) group by two‐way ANOVA followed by Tukey's post hoc test, *n* = 8 in each group). (D, E) mRNA levels of IL‐1β, IL‐6, TNF‐α, IL‐10, and TGF‐β after injection of miR‐26a‐5p agomir. Statistical significance of mean differences was determined with the Tukey's multiple comparisons test.

First, we adopted a mouse model of inflammatory pain by subcutaneously injecting 20 μL of CFA into the hind paw of mice. miR‐26a‐5p agomir was then injected to mice for 8 h after modeling by two methods of injection: intrathecal injection in subarachnoid space and subcutaneous injection in mice hind paw (Figure 1A). The results showed that, on the first day after modeling, both intrathecal injection and subcutaneous injection of miR‐26a‐5p agomir had a significant antinociceptive effect compared with the CFA modeling group (Figure 1B,C). However, subcutaneous injection of miR‐26a‐5p agomir in the mice hind paw showed apparently weak analgesia than intrathecal injection via mechanic threshold and thermal latency test. Moreover, although the antinociceptive effect of subcutaneous injection was still existed on day 3 compared with the CFA model group, there was no significant statistical difference. Compared with subcutaneous injection, intrathecal injection still maintained a significant increase of mechanical threshold and thermal latency at the 7th day, although the analgesic effect has a marked decline from the 3rd day. These results suggested that miR‐26a‐5p agomir can produce a decent analgesia in a short term after a single intrathecal or subcutaneous injection, while intrathecal injection could produce a more potent and long‐lasting analgesia effect than subcutaneous injection. We took the hind paw tissue of the mice one day after intrathecal or subcutaneous injection of miR‐26a‐5p agomir (Figure 1A) to examine the changes of inflammation‐related cytokines after different administration methods. It can be clearly seen that both intrathecal and local subcutaneous injection of miR‐26a‐5p agomir and the levels of inflammatory cytokines IL‐1β, IL‐6, and TNF‐α in mice hind paws were significantly downregulated (Figure 1D), while antiinflammatory cytokines IL‐10 and TGF‐β were significantly upregulated (Figure 1E). In addition, intrathecal injection of miR‐26a‐5p agomir appears to have a more pronounced increase in antiinflammatory cytokines compared with local subcutaneous injection (Figure 1E). These results suggest that miR‐26a‐5p can reduce CFA‐induced inflammatory pain and suppress inflammation in mice paw tissue by a single intrathecal or subcutaneous injection.

### 3.2 miR‐26a‐5p can significantly alleviate complete Freund's adjuvant‐induced peripheral inflammation and neuroinflammation at the spinal cord level

**FIGURE 2:**
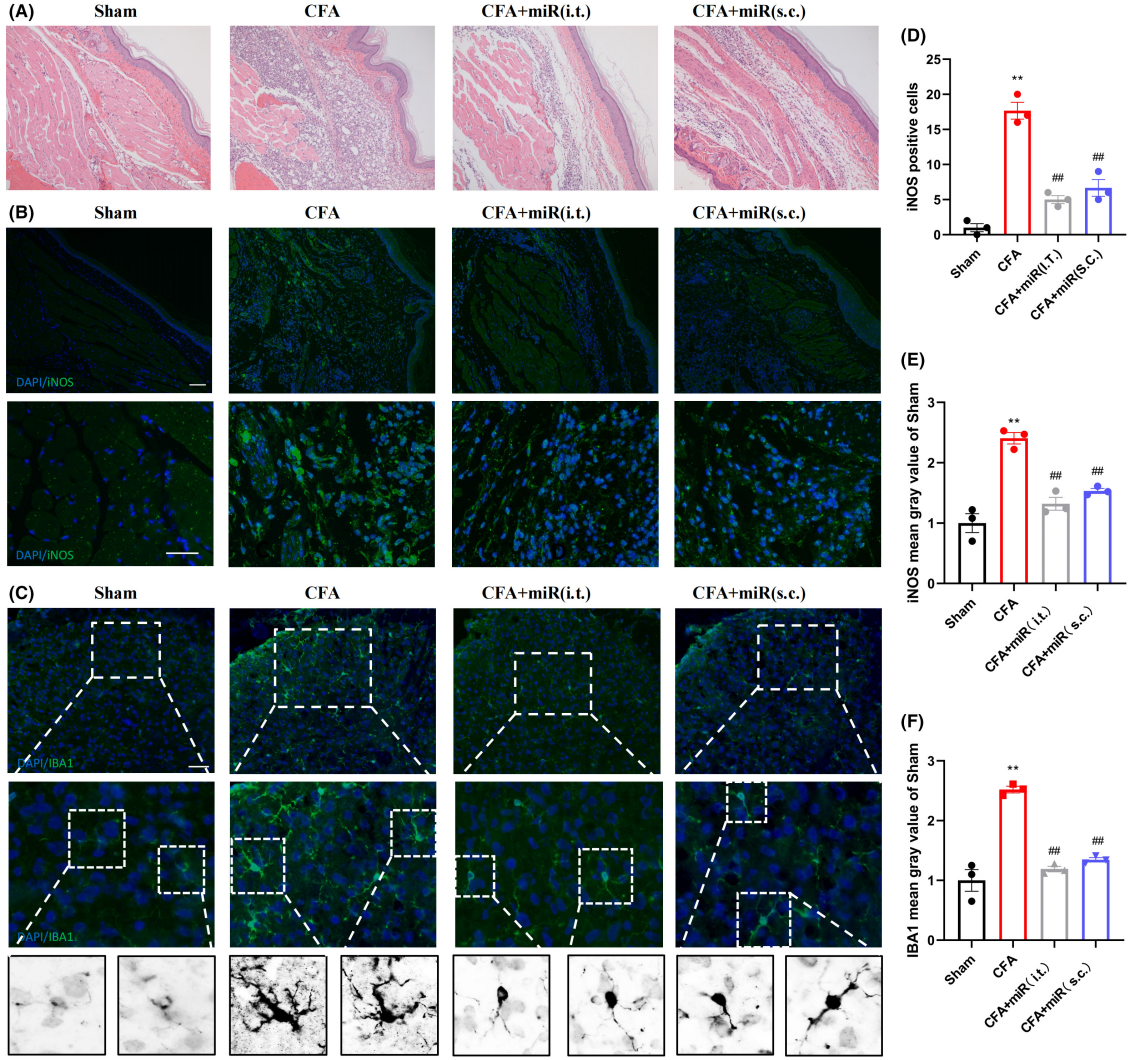
miR‐26a‐5p significantly alleviates complete Freund's adjuvant‐induced peripheral inflammation and neuroinflammation by different administration modes. (A) HE staining after injection of miR‐26a‐5p agomir. (B) Immunofluorescent study revealed iNOS‐positive cells (green) in the hind paw subcutaneous tissue of CFA‐induced inflammatory pain mice in 10 × (scale bar: 100 μm) and 20 × (scale bar: 50 μm) fluorescence microscope. The blue spots are DAPI nuclear staining. (C) Immunofluorescent study revealed IBA1‐postive microglia (green) in the spinal dorsal horn of CFA‐induced inflammatory pain mice (scale bar: 100 μm). The blue spots are DAPI nuclear staining. (D) Quantification showing the number of iNOS‐positive cells was significantly increased in the CFA group compared with the sham, CFA/miR(i.t.) and CFA/miR(s.c.) groups. (E, F) Quantification showing the mean gray value of iNOS‐ and IBA1‐positive cells were significantly increased in the CFA group, compared with sham, CFA/miR(i.t.) and CFA/miR(s.c.) group. Data are represented as mean ± sem. **p < 0.01. ##*p* < 0.01.

Next, we performed HE staining on the paw tissue. The images of HE staining exhibited that the subcutaneous tissue of the hind paw has obvious inflammatory infiltration and tissue structure damage after modeling (Figure 2A). After intrathecal or local subcutaneous injection of miR‐26a‐5p agomir, the inflammatory infiltration of subcutaneous tissue was significantly alleviated. Similar to the analgesic effect, intrathecal injection reduces inflammatory infiltration better than subcutaneous administration. We then analyzed iNOS‐positive cells in mice hind paw subcutaneous tissue, and we found that iNOS expression in cells were significantly elevated after CFA modeling (Figure 2B). The number of iNOS‐positive cells in the subcutaneous tissue of the mice hind paw was significantly decreased in both miR‐26a‐5p agomir administration groups (Figure 2D,E). In the dorsal horn of the spinal cord, we analyzed IBA1‐positive microglia, and we can also see that after CFA caused inflammatory pain in mice, the microglia in the dorsal horn of the L4‐5 spinal cord were significantly activated (Figure 2C). After intrathecal and local subcutaneous injection of miR‐26a‐5p agomir, the number of activated microglia decreased markedly (Figure 2C,F). Taken together, whether intrathecal injection or local subcutaneous injection of miR‐26a‐5p agomir can significantly alleviate both spinal and local subcutaneous inflammation of mice after CFA modeling. Comparing the two methods of administration, intrathecal injection showed better antiinflammatory effect in spinal cord and paw as well as more effective and long‐lasting based on our mechanical and thermal pain behavioral tests (Figure 1B,C).

### 3.3 Wnt5a is a direct target gene of miR‐26a‐5p and is expressed in neurons and microglia in the dorsal horn of the spinal cord

**FIGURE 3:**
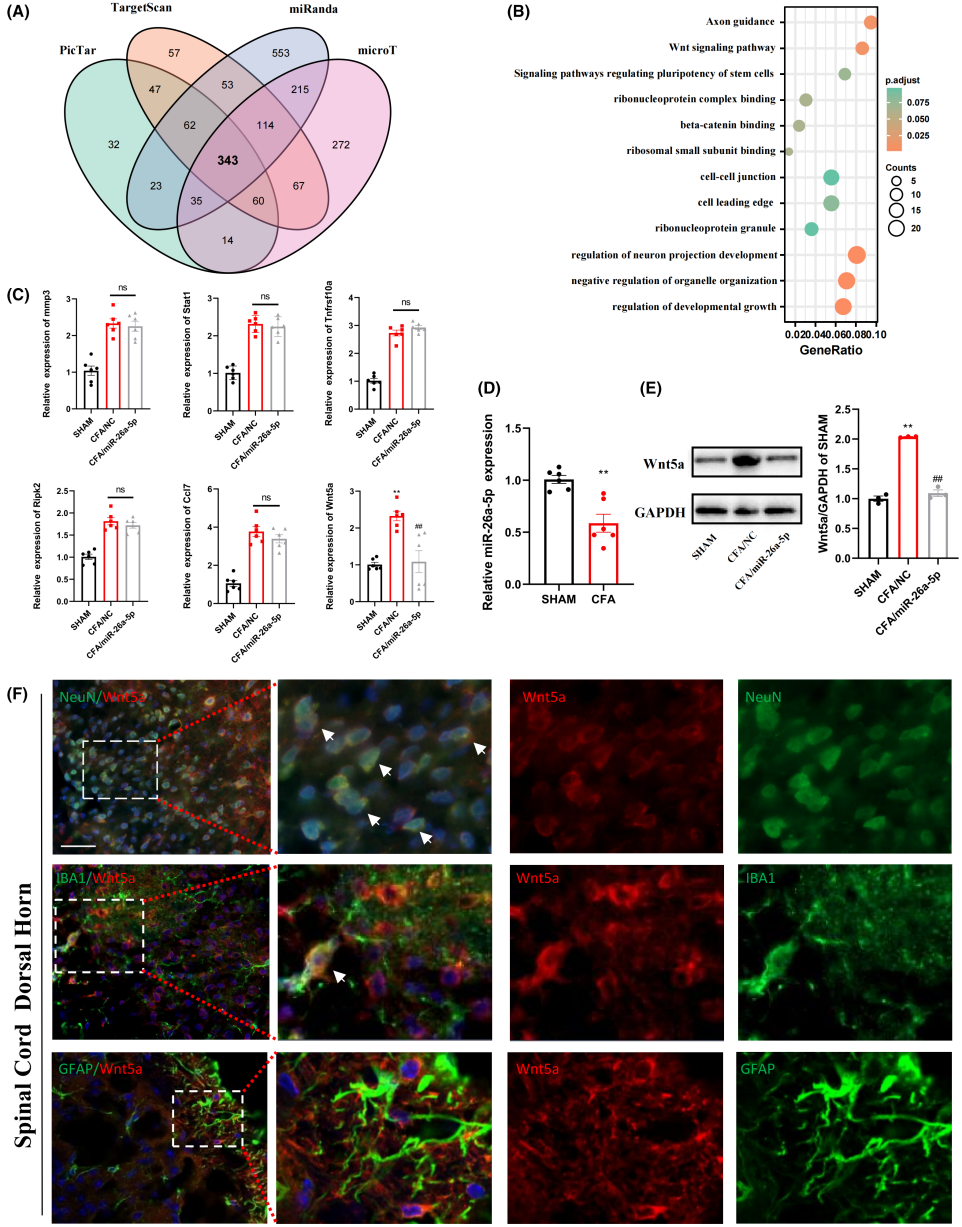
miR‐26a‐5p directly targets Wnt5a and the cellular localization of Wnt5a in the dorsal horn of the spinal cord. (A) Venn diagram showing target mRNAs for miR‐26a‐5p from four databases. (B) Gene ontology enrichment analysis of target genes for miR‐26a‐5p. (C) mRNA level of gene associated with inflammation in Sham, CFA/NC, and CFA/miR‐26a‐5p groups. (D) Relative expression of miR‐26a‐5p in Sham and CFA groups. (E) Protein level of Wnt5a in Sham, CFA/NC, and CFA/miR‐26a‐5p groups. The CFA/miR‐26a‐5p group decreased significantly compared with CFA/NC group, *n* = 6 mice/group. Data are represented as mean ± sem. **p < 0.01. ##*p* < 0.01. (F) Immunofluorescent study revealed Wnt5a (red) localized within GFAP astrocyte/IBA1 microglia/NeuN neuron (green) in the L4‐5 spinal dorsal horn of CFA‐induced inflammatory pain mice. Wnt5a co‐localized mostly with NeuN neuron. Some appeared in the IBA1 microglia. Few could be detected in the GFAP astrocyte. The blue spots are DAPI nuclear staining (scale bar: 50 μm).

We used different miRNA target gene prediction databases, including miRanda, DIANA‐microT, TargetScan, and PicTar, to predict possible target genes of miR‐26a‐5p and the intersection of the four data obtained 343 common predicted target genes. Then, we performed gene ontology and KEGG pathway analysis on these 343 target genes (Figure 3A,B), in which Wnt signaling pathway is highly enriched. We also found that the Wnt5a gene exists in several pain‐related and highly enriched entries (axon guidance, regulation of neuron projection development, etc.). Besides, we selected genes associated with inflammation from predicted pathways for validation, including Mmp3, Stat1, Tnfrsf10a, Ripk2, and Ccl7. We performed qPCR on these genes and found that Mmp3, Stat1, Tnfrsf10a, Ripk2, and Ccl7 did not change significantly after intrathecal injection of miR‐26a‐5p agomir, while the mRNA expression of Wnt5a decreased significantly (Figure 3C). We detected the expression of miR‐26a‐5p in the L4‐5 segment of the spinal cord on the third day after modeling, and miR‐26a‐5p was significantly downregulated on the third day after modeling (Figure 3D). Then, we analyzed the changes of Wnt5a protein expression, and we can see that after the injection of CFA to induce inflammatory pain in mice, the protein expression of Wnt5a in the L4‐5 segment of spinal cord increased significantly. After intrathecal injection of miR‐26a‐5p agomir, Wnt5a expression levels were significantly reversed (Figure 3E). Wnt5a is highly expressed in the dorsal horn of the spinal cord after pain, but in the CFA‐induced inflammatory pain model, the specific cellular location of Wnt5a overexpression remains unclear.^38^, ^39^, ^40^, ^41^ We took the dorsal horn of the L4‐5 segment of the spinal cord for analysis on the third day of CFA‐induced inflammatory pain. We found that Wnt5a expression could be found in NeuN‐positive and IBA1‐positive cells, but not in GFAP‐positive cells (Figure 3F). Therefore, we selected Wnt5a for further study, and the elevation of Wnt5a were mainly in neurons and microglia in the spinal dorsal horn of mice with CFA‐induced inflammatory pain.

### 3.4 miR‐26a‐5p inhibits the upregulation of Wnt5a/CaMKII/NFAT pathway after CFA‐induced inflammatory pain

**FIGURE 4:**
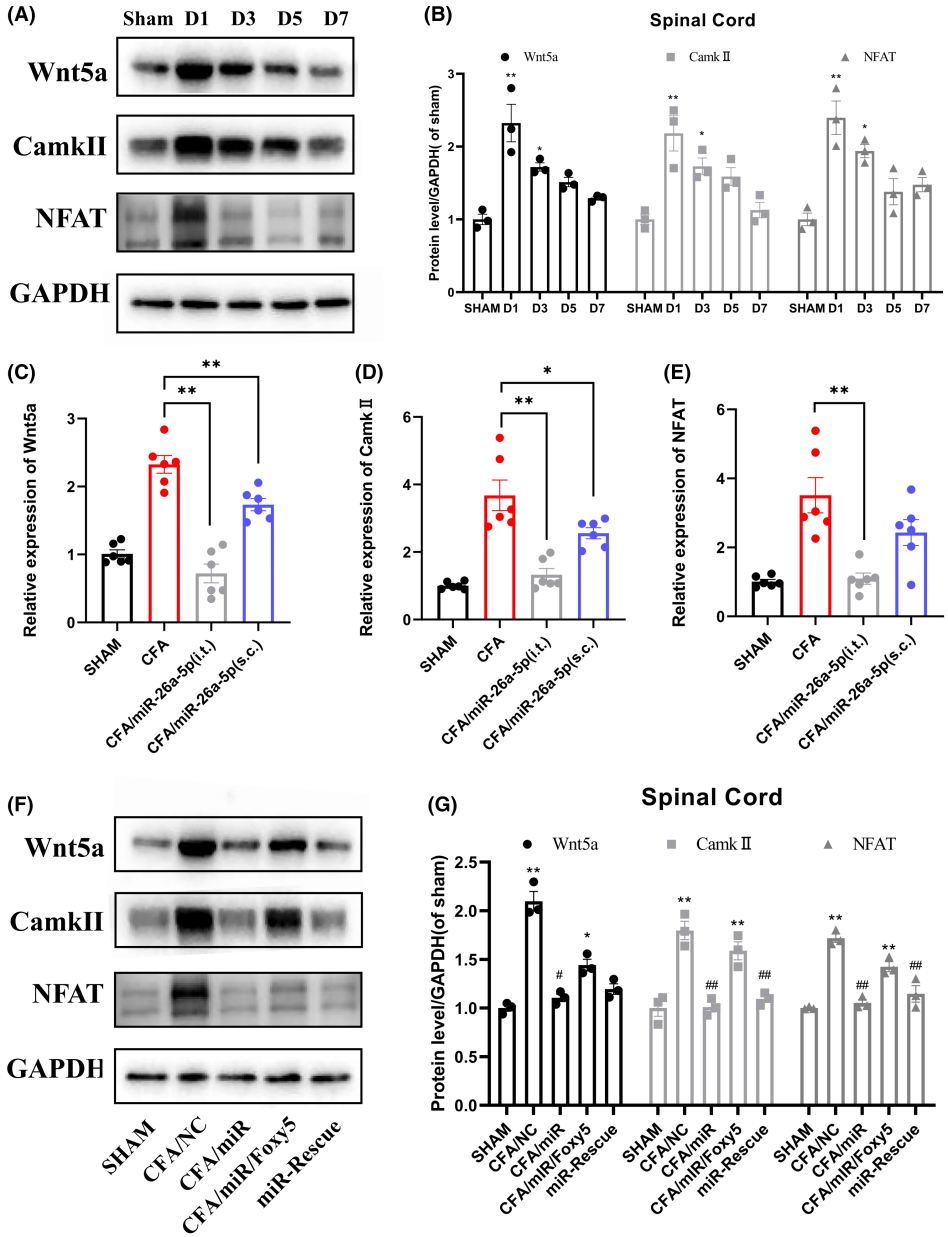
miR‐26a‐5p inhibits the upregulation of Wnt5a/CaMKII/NFAT pathway after CFA‐induced inflammatory pain. (A, B) Western blot was used to assess the level of Wnt5a, CaMKII, and NFAT at day 1, 3, 5, and 7 after CFA modeling. Data are expressed as fold change compared to the sham group. (C–E) mRNA levels of Wnt5a(C), CaMKII (D), and NFAT(E) after injection of miR‐26a‐5p agomir. (F, G) Western blot was used to assess the level of Wnt5a, CaMKII, and NFAT after Foxy‐5 reverse and miR‐26a‐5p rescue. Representative immunoblots and quantification showing Foxy‐5 reversed the effect of miR‐26a‐5p, while intrathecal injection of miR‐26a‐5p two days after Foxy‐5 again rescued the effect of Foxy5. Data are expressed as fold change compared to the sham group. Data are represented as mean ± sem. *p < 0.05, **p < 0.01. #*p* < 0.05, ##*p* < 0.01.

It has been previously reported that the noncanonical Wnt signaling pathway is partially mediated by Wnt5a and causes an elevation of downstream CaMKII expression, ultimately affecting the expression of the transcription factor NFAT.^42^, ^43^, ^44^, ^45^ Therefore, we examined the protein expression of Wnt5a, CaMKII, and NFAT at the spinal cord of different time points after CFA‐induced inflammatory pain (Figure 4A,B). It can be clearly seen that the expression of Wnt5a, CaMKII, and NFAT increased significantly on the first day and continued to the fifth day or even longer, and the changes of Wnt5a, CaMKII, and NFAT were basically follow the same style. Next, we also used different miR‐26a‐5p agomir administration methods to observe the expression changes of noncanonical Wnt signaling molecules Wnt5a, CaMKII, and NFAT. mRNA levels of Wnt5a, CaMKII, and NFAT were significantly decreased regardless of intrathecal or local subcutaneous injection (Figure 4C–E). Consistent with the previous comparison of the two administration methods (Figure 1; Figure 2), intrathecal injection has a more significant downregulation effect on noncanonical Wnt signaling molecules. We then used Foxy5, a mimetic peptide of Wnt5a, to reverse the effect of miR‐26a‐5p and elucidate the relationship between Wnt5a, CaMKII, and NFAT. After intrathecal injection of miR‐26a‐5p agomir, the protein expression of Wnt5a, CaMKII, and NFAT decreased significantly(Figure 4F,G). Then, we injected 10 μL Foxy5 intrathecally, and the expression of Wnt5a, CaMKII, and NFAT were reversed to varying degrees, and the three proteins both showed significant increase. After that, we injected miR‐26a‐5p agomir again intrathecally, Foxy5‐induced upregulation of Wnt5a, CaMKII, and NFAT was rescued again. In conclusion, miR‐26a‐5p significantly inhibited the expression of Wnt5a, CaMKII, and NFAT in noncanonical Wnt signaling pathways after CFA‐induced inflammatory pain.

### 3.5 miR‐26a‐5p suppresses LPS‐induced inflammation in BV2 cells in vitro through noncanonical Wnt signaling

**FIGURE 5:**
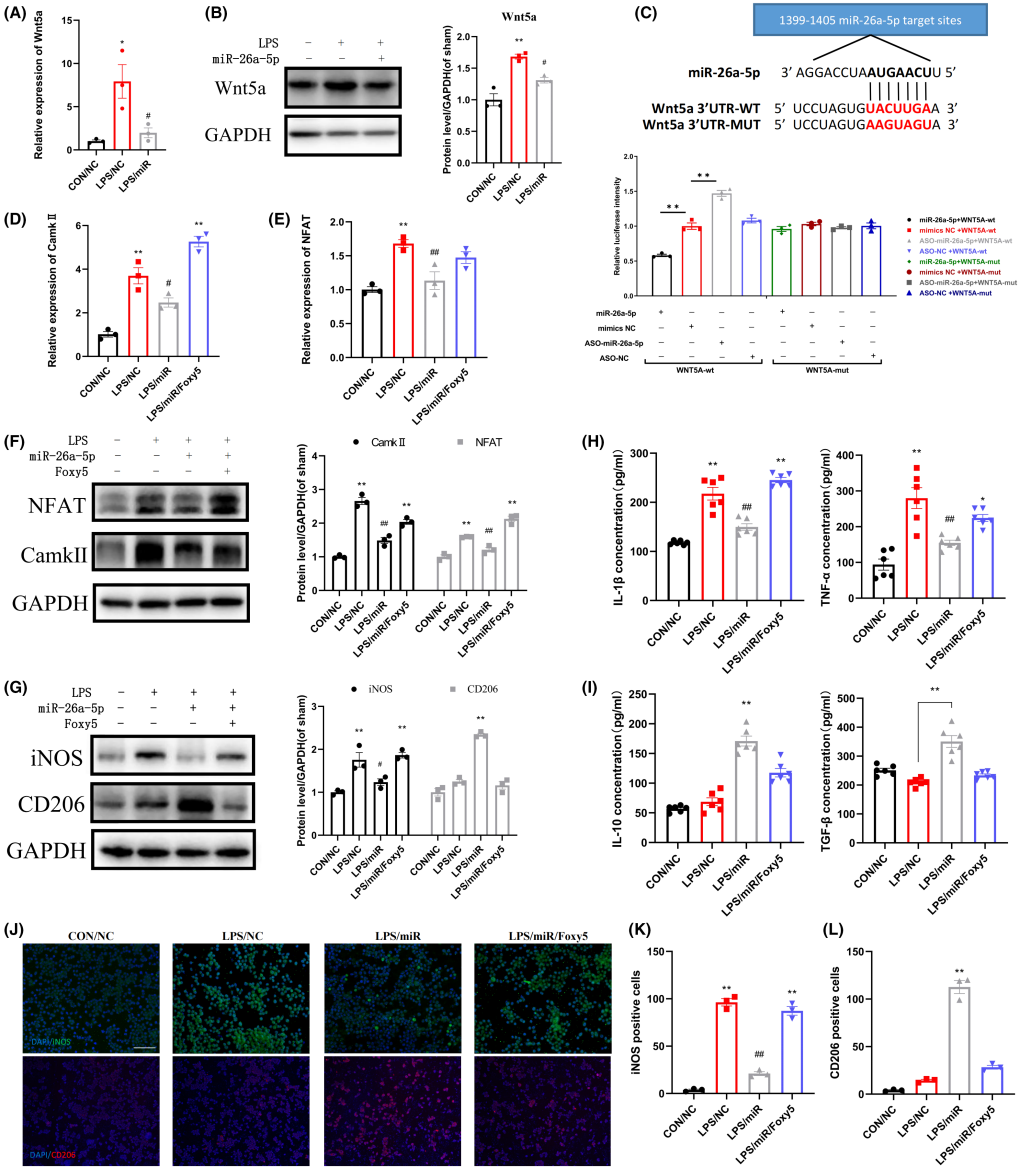
miR‐26a‐5p suppresses LPS‐induced inflammation in BV2 cells in vitro through noncanonical Wnt signaling. (A, B) mRNA(A) and protein(B) levels of Wnt5a in CON/NC, LPS/NC, and LPS/miR groups. The LPS/miR group decreased significantly compared with LPS/NC group. (C) The binding and mutant sequence of miR‐26a‐5p in Wnt5a and Luciferase reporter assays in BV2 cells after co‐transfection of cells with the wild type or mutant 3’‐UTR of Wnt5a and the miR‐26a‐5p, NC mimics or ASO‐miR‐26a‐5p, ASO‐NC. (D–F) mRNA(D, E) and protein(F) levels of CaMKII and NFAT in CON/NC, LPS/NC, LPS/miR, and LPS/miR/Foxy5 groups. (G) Western blot was used to assess the level of iNOS and CD206 of BV2 cells. Data are expressed as fold change compared to the sham group. (H, I) The protein levels of IL‐1β, TNF‐α(H), IL‐10, and TGF‐β(I) in cell culture supernatant were tested by ELISA. (J) Immunofluorescent study revealed iNOS‐positive (green) and CD206‐positive (red) BV2 cells. The blue spots are DAPI nuclear staining (scale bar: 100 μm). (K, L) Quantification showing the number of iNOS‐positive(K) and CD206‐positive(L) BV2 cells. Data are represented as mean ± sem. *p < 0.05, **p < 0.01. #*p* < 0.05, ##*p* < 0.01.

Next, we used BV2 cells to verify the mechanism in vitro. After 48 h of transfection with miR‐26a‐5p, we stimulated the cells with LPS (500 ng/mL) for 24 h. First, we detected the expression of downstream molecules of noncanonical Wnt signaling pathway in cells after different treatments. It can be seen that the expression of Wnt5a was significantly increased at both mRNA and protein levels after LPS stimulation of BV2 cells for 24 h, while the LPS/miR group was significantly downregulated compared with the LPS/NC group (Figure 5A,B). After LPS stimulation of BV2 cells, the expressions of CaMKII and NFAT, downstream molecules of Wnt5a‐mediated noncanonical Wnt signaling, were also significantly increased at both mRNA and protein levels. Likewise, the expressions of CaMKII and NFAT were downregulated in the LPS/miR group compared with the LPS/NC group. This downregulation can be reversed to varying degrees by Foxy5 (Figure 5D–F). Then, we validated the direct binding of miR‐26a‐5p and Wnt5a 3’‐UTR using dual‐luciferase reporter assay in BV2 cells (Figure 5C). The results showed that the Renilla luciferase activity of pmirGLO‐Wnt5a‐WT transfected cells decreased by about 50% in miR‐26a‐5p co‐transfected cells compared with mimic‐NC co‐transfected cells; however, expression increased by about 50% in ASO‐miR‐26a‐5p co‐transfected cells compared with NCs. In addition, luciferase levels of pmir‐GLO‐Wnt5a‐Mu transfected cells did not change in miR‐26a‐5p or ASO‐miR‐26a‐5p co‐transfected cells. These results indicate that miR‐26a‐5p can directly target the 3’‐UTR of Wnt5a and regulate noncanonical Wnt signaling.

We then analyzed the proportion of M1‐ and M2‐type microglia. It can be seen that the number of iNOS fluorescence‐positive cells in BV2 cells in the LPS/NC group increased significantly, and the number of positive cells in the LPS/miR group was significantly lower than that in the LPS/NC group (Figure 5J,K). After the cells were stimulated by Foxy5(1 μg/μL), a peptide mimetic of Wnt5a, the number of iNOS‐positive cells increased significantly. We also observed that CD206 expression was significantly increased in the LPS/miR group, and this effect was reversed by Foxy5 (Figure 5J,L). We also extracted the proteins of cells with different treatments, and we could see that the expression of iNOS was significantly increased after LPS stimulation, while it was downregulated in the LPS/miR group. After treating cells with Foxy5, the expression of iNOS was significantly upregulated again. The protein expression of CD206 was significantly upregulated in the LPS/miR group compared with the LPS/NC group, and this effect was reversed by Foxy5 (Figure 5G). We then used Elisa to detect inflammatory factors in cell culture supernatants. It can be seen that after stimulating BV2 cells with LPS for 24 h, the levels of IL‐1β and TNF‐α in the supernatant were significantly upregulated, while the LPS/miR group was significantly decreased compared with the LPS/NC group. This decline was reversed 24 h after Foxy5 stimulation of BV2 cells (Figure 5H). In addition, we also found that the expression of antiinflammatory factors TGF‐β and IL‐10 was significantly increased in LPS/miR group compared with LPS/NC group, and this effect was also reversed by Foxy5(Figure 5I). In summary, miR‐26a‐5p suppresses LPS‐induced inflammation in BV2 cells in vitro through noncanonical Wnt signaling.

### 3.6 miR‐26a‐5p attenuates nociception induced by inflammation and suppresses spinal neuroinflammation via noncanonical Wnt signaling

**FIGURE 6:**
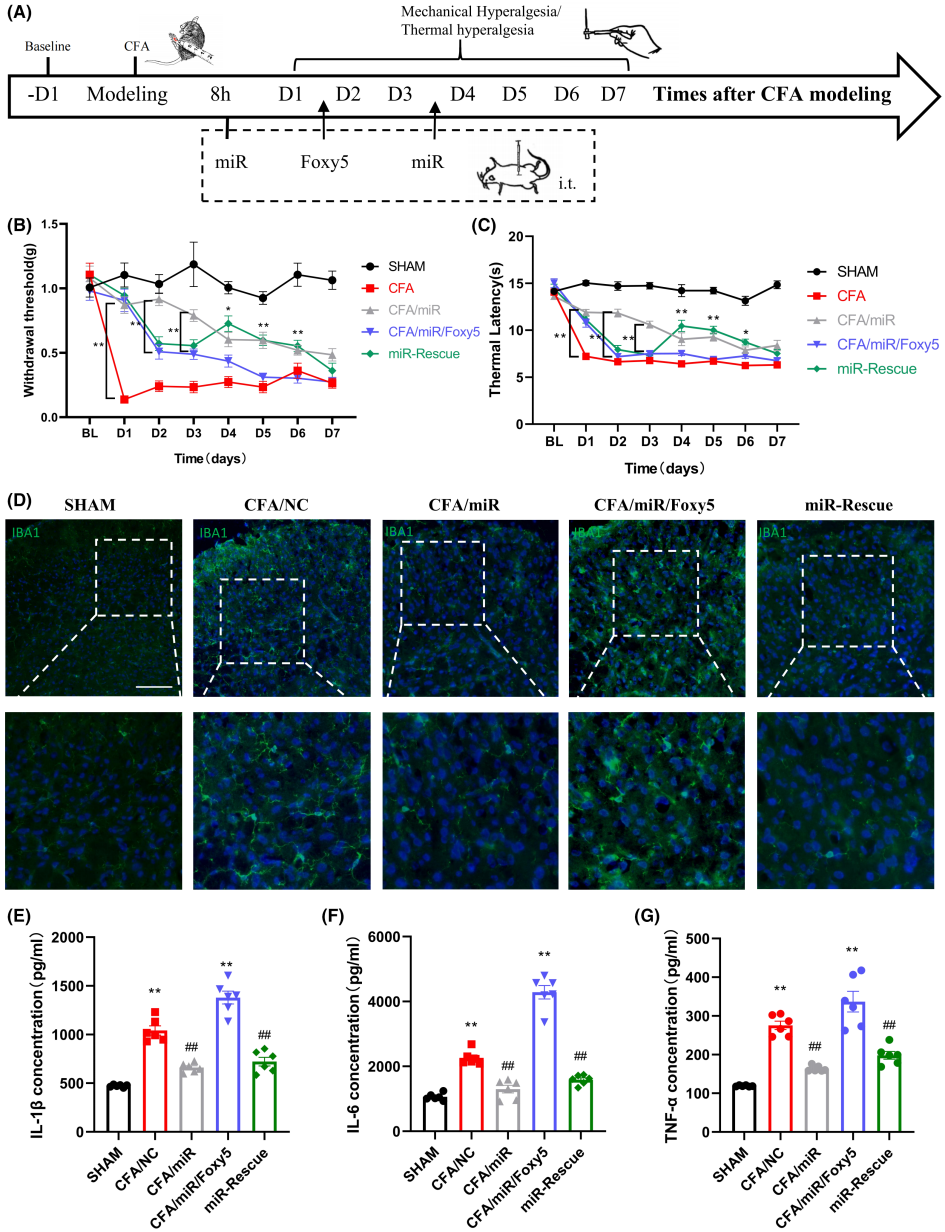
miR‐26a‐5p attenuates nociception induced by inflammation and suppresses spinal neuroinflammation via noncanonical Wnt signaling. (A) Experimental protocol for establishing complete Freund's adjuvant‐induced inflammatory pain mouse model, different drug interventions, and pain behavioral test. (B) 50% paw withdraw threshold (PWT) of left hind paw of mice in different treatment groups mice. At days 2 and 3, the paw withdrawal thresholds (WTs) of CFA/miR group were significantly higher than those of CFA/miR/Foxy5 group after Foxy5 reverse at day 1 (*p < 0.05, **p < 0.01 compared with CFA/miR group by two‐way ANOVA followed by Tukey's post hoc test, *n* = 8 in each group). (C) Thermal latency(s) of left hind paw of mice in different treatment groups. At days 4, 5, and 6, the paw thermal latency(s) of miR‐Rescue group was significantly higher than those of CFA/miR/Foxy5 group after miR‐26a‐5p rescue at day 3 (*p < 0.05, **p < 0.01 compared with CFA/miR/Foxy5 group by two‐way ANOVA followed by Tukey's post hoc test, *n* = 8 in each group). (D) Immunofluorescent study revealed IBA1 microglia (green) in the L4‐5 spinal dorsal horn of CFA‐induced inflammatory pain mice. The blue spots are DAPI nuclear staining (scale bar: 100 μm). (E–G) The protein levels of IL‐1β (E), IL‐6 (F), and TNF‐α (G) in the L4‐L5 dorsal spinal cord of mice were tested by ELISA. *n* = 6 mice for each group. Data are represented as mean ± sem. **p < 0.01. ##*p* < 0.01.

Next, we injected miR‐26a‐5p agomir intrathecally 8 h after CFA subcutaneous injection, injected Foxy5 intrathecally post‐CFA injection Day 1, and intrathecally injected miR‐26a‐5p agomir again post‐CFA injection Day 3 (Figure 6A). We then observed the changes of mechanical threshold and thermal latency in the mice hind paw after different treatments. It can be clearly seen that on the first day after intrathecal injection of miR‐26a‐5p agomir, the mechanic threshold and thermal latency of mice were significantly increased (Figure 6B,C). After the behavioral test post CFA injection Day 1, we intrathecally injected Foxy5, and it could be seen that there was a significant decrease in the mechanic threshold and thermal latency of the mice hind paw post‐CFA injection days 2 and 3. Then, we injected miR‐26a‐5p agomir intrathecally again post CFA injection Day 3, and we could see that the mechanic threshold and thermal latency of the hind paw were rescued to varying degrees post CFA injection Day 4, and this effect continued until Day 6. However, the analgesic effect was not as significant as that of intrathecal injection of miR‐26a‐5p agomir 8 h after CFA modeling. As Figure 2C; Figure 6D showed, microglia in the dorsal horn of the spinal cord were significantly activated after CFA‐induced inflammatory pain, and this activation was alleviated after our intrathecal injection of miR‐26a‐5p agomir. We then explored whether Foxy5 could reverse the effects of miR‐26a‐5p. After intrathecal injection of Foxy5, the microglia in the dorsal horn of the mouse spinal cord significantly activated compared with the miR‐26a‐5p injection group (Figure. 6D). Likewise, we injected an additional miR‐26a‐5p agomir intrathecally, and Foxy5‐induced microglial activation was rescued. At the same time, we also used ELISA to detect pro‐inflammatory factors in the spinal cord. The expression of IL‐1β, IL‐6, and TNF‐α were all upregulated to varying degrees in the spinal cord of the L4‐5 segment after intrathecal injection of Foxy5. When we injected miR‐26a‐5p agomir to rescue the effect of Foxy5, the levels of IL‐1β, IL‐6, and TNF‐α were downregulated again. This downregulation was not as pronounced as that of the first intrathecal injection of miR‐26a‐5p agomir in TNF‐α, which is also consistent with the results of our behavioral tests (Figure 5E–G).

## 4 DISCUSSION

**FIGURE 7:**
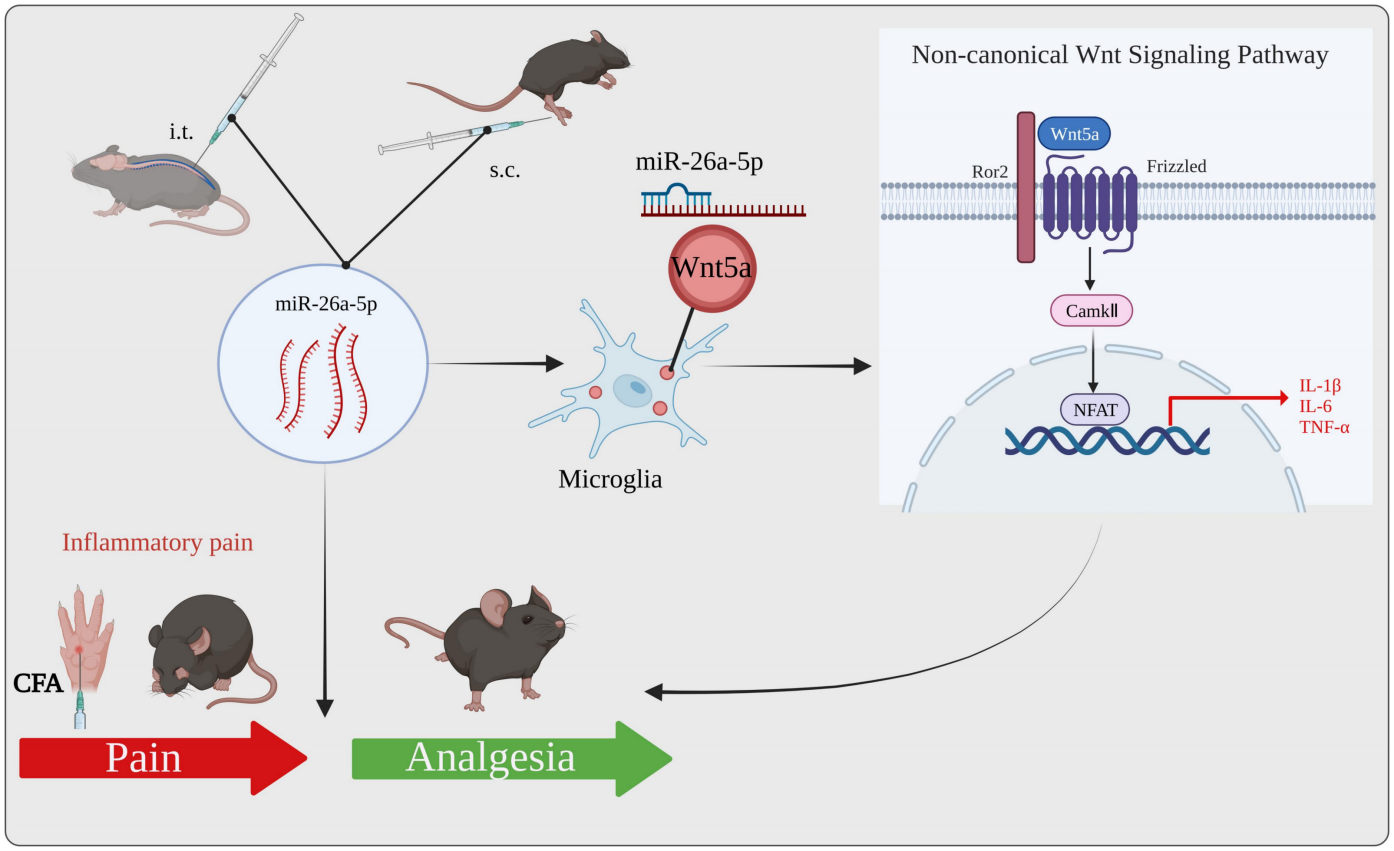
miR‐26a‐5p alleviates CFA‐induced chronic inflammatory hyperalgesia through Wnt5a/CaMKII/NFAT signaling in Mice. miR‐26a‐5p regulated Wnt5a/CaMKII/NFAT, a noncanonical Wnt signaling, involved in analgesia partly through antineuroinflammation, suggesting a pain‐alleviating effect via noncanonical Wnt signaling pathway in CFA‐induced inflammatory pain model in vivo and vitro.

In current study, we found that miR‐26a‐5p had a significant alleviation effect on CFA‐induced inflammatory pain. Our results found that both intrathecal and subcutaneous local injection of miR‐26a‐5p agomir could alleviate inflammatory pain via antiinflammation in spinal cord and hind paw. Intrathecal injection could produce longer pain relief period, which lasting to at least 7 days after miR‐26a‐5p injection. Moreover, both intrathecal and local application miR‐26a‐5p can significantly reduce the inflammatory factors level, alleviate inflammatory infiltration, and reverse the inflammation‐induced subcutaneous tissue lesion of the mice hind paw. Furthermore, we found that two administration methods could alleviate activation of macrophages and microglia in subcutaneous tissue of paw and dorsal horn of the spinal cord, respectively. We also found that miR‐26a‐5p could reverse the M1/M2 proportion after LPS‐stimulated BV2 cells. Then, our in vitro and in vivo study suggested that miR‐26a‐5p could target Wnt5a to decrease the IL‐1β, TNF‐α, and IL‐6 through regulating CamK II and NAFT. Taken together, our study confirmed that miR‐26a‐5p could relief inflammation through intrathecal or subcutaneous local injection. We also demonstrated that the analgesia of intrathecal injection miR‐26a‐5p is produced by targeting Wnt5a, a noncanonical Wnt signaling pathway, to regulate the inflammation of spinal cord and hindpaw (Figure 7).

Myeloid cells, especially microglia and macrophages, is the pivotal cells during the pathophysiologic process of inflammatory pain, which is closely related with many potential mechanisms, including activation of ion channels, synaptic plasticity, and central sensitization, in the inflammatory pain onset, maintenance, and lasting.^46^ Our results found that the activated cell number of microglia in spinal cord and macrophages in hindpaw have been significantly decreased by miR‐26a‐5p injection, which is consistent with previous reports.^30^ In the current study, we adopted two different methods to inject miR‐26a‐5p, intrathecal injection and subcutaneous injection in hindpaw, which not only aim to compare the efficacy of different injection methods, but try to screen a better method which may help to avoid the potential complications of intrathecal injection such as bleeding, infection,^47^ for future developing optimal therapy. Although subcutaneous injection could reduce activation of spinal microglia, which may result from local injection of miR‐26a‐5p inhibited the inflammatory stimuli, the subcutaneous injection could only provide a one‐day pain‐relieving period. Thus, we selected the intrathecal injection in the following parts to investigate the potential mechanism of miR‐26a‐5p.

Spinal microglia are considered to be a significant central system resident immune cell that plays a crucial role in the inflammatory response.^48^ The pathogenesis of inflammatory pain is known to be closely related to the release of pro‐inflammatory mediators and activation of microglia.^14^ It has been previously reported that microglia are key initiators of the development of inflammatory pain.^49^ Activated microglia trigger the release of pro‐inflammatory cytokines, such as IL‐1β, IL‐6, and TNF‐α,^50^ which can further activate microglia.^51^ In our study, microglia in the dorsal horn of the spinal cord significantly activated after CFA‐induced inflammatory pain. Also, microglia activation leads to enhancement of pro‐inflammatory cytokines and maintains central sensitization and inflammatory pain.^52^ Therefore, inhibition of microglia activation may be an important target for inflammatory pain. In our study, we found that miR‐26a‐5p could inhibit the activation of spinal dorsal horn microglia in vivo in the state of inflammatory pain, along with the same downregulation of pro‐inflammatory factors, and it could also reverse M1/M2 proportion of BV2 cells under LPS stimulation in vitro. In previous studies, miR‐26a‐5p has shown a promising biological effects and therapeutic potential in various diseases. Many investigations reported the anticancer effect of miR‐26a‐5p through inducing apoptosis, inhibiting proliferation, and decreasing metastasis of different cancer cells.^53^, ^54^, ^55^ Apart from cancers, miR‐26a‐5p also exhibit protection in cardiovascular diseases. It could protect against myocardial ischemia/reperfusion injury by regulating PTEN/PI3K/AKT signaling pathway^56^ and also alleviates myocardial infarction by regulating ATG12‐mediated autophagy in cardiomyocytes.^57^ In neurological diseases, miR‐26a‐5p could target DYRK1A to inhibits Tau phosphorylation and Aβ accumulation, which ameliorated the cognitive dysfunction, in Alzheimer's disease mice.^58^ More importantly, miR‐26a‐5p presented favorable antiinflammatory effect in several disease model. In cerebral ischemic/reperfusion brain injury, miR‐26a‐5p could target CDK6 to induce microglial apoptosis^59^ and also could target NRSF to reduce neuroinflammation,^60^ which both alleviated the brain injury. miR‐26a‐5p also regulates inflammation through other target genes. For example, miR‐26a‐5p alleviated acute lung injury by targeting TLR4 to suppress inflammation and apoptosis,^29^ and it decreased inflammation in diabetic nephropathy by targeting CHAC1/NF‐κB pathway.^61^ In our study, we also found that miR‐26a‐5p significantly downregulated the inflammatory cytokines secreted from microglia by targeting Wnt5a in vivo and in vitro, which are closely related with the significant alleviation of nociceptive sensations in inflammatory pain mice model. We chose the BV2 cell line here to create an in vitro model of microglia. Primary microglia is useful in vitro model for mechanistic studies because they recapitulate most of the known physiological activities of microglia in vivo, including phagocytosis, migration, and the release of pro‐inflammatory cytokines and chemokines upon stimulation.^62^ In addition to primary microglia, microglia‐like cell lines have been created and widely used to examine the mechanistic details of microglial function, including mouse immortalized BV2 cells. RNA‐sequencing analysis reveals that most primary microglia‐specific immune functions and pathways are preserved in BV2 cells.^63^ However, although immortalized cells are easy to replicate and maintain in culture, their validity as sufficient substitutes for primary microglia is somewhat controversial.^64^ Functionally, microglial cell lines share similarities with primary microglia, but differences exist in secretion and gene expression upon LPS stimulation.^65^, ^66^ In addition, although both BV2 cells and primary microglia express Iba1, a microglia marker, BV2 cells showed much less induction of some pro‐inflammatory genes and much lower levels of cytokine secretion by LPS compared with primary microglia.^65^, ^66^ Therefore, the BV2 cell line has certain limitations as an in vitro model of microglia.

Wnt5a, a ligand of the noncanonical Wnt signaling pathway, was previously reported to be expressed in spinal dorsal horn and DRG neurons of naive mice.^39^ Previous studies have shown that Wnt5a is upregulated in various models of chronic pain, including inflammatory pain,^40^ neuropathic pain,^39^ chronic post‐thoracotomy pain,^67^ etc., and may play a key role in the development of pain.^68^ In different types of pain, Wnt5a‐mediated activation of noncanonical Wnt pathways often leads to microglia activation and neuroinflammation.^69^ In a rat model of CCI‐induced neuropathic pain, Zhang et al. demonstrated that intrathecal injection of IWP‐2, a Wnt signaling pathway inhibitor, significantly inhibited microglial activation in the spinal cord and reduced mechanical allodynia and thermal hyperalgesia.^70^ Additionally, another inhibitor, IWR‐1‐endo, was also shown to alleviate vincristine‐induced neuropathic pain by inhibiting microglial activation in the spinal cord.^71^ What's more, Wang et al. demonstrated that crocin, a blocker of Wnt5a receptor, significantly alleviated mechanical allodynia in AIA rats by inhibiting the spinal cord Wnt5a signaling pathway and glial cell activation.^72^ Noncanonical Wnt pathways (categorized according to their downstream involvement in β‐catenin) play critical roles also by regulating synaptic transmission, plasticity, and intraepidermal nerve fiber density.^45^ It has been previously reported that Wnt5a is highly expressed in different types of cells in the spinal cord and DRG. Wang et al. found that Wnt5a is mainly expressed in neurons in the dorsal horn of the spinal cord of rats with adjuvant arthritis (AIA).^72^ In a rat model of chronic post‐thoracotomy pain, Zhu et al. showed that increased Wnt5a expression mainly colocalizes with peptidergic CGRP and non‐peptidergic Isolectin B4 (IB4) neurons in the DRG.^67^, ^73^ In our study, we found that Wnt5a mainly colocalized with neurons and microglia in the dorsal horn of the spinal cord of mice with inflammatory pain. Previous studies have also shown that calcium/calmodulin‐dependent protein kinase II (CaMKII) is a multifunctional serine/threonine kinase essential for nociceptive transmission and processing.^74^, ^75^ Wnt5a can enhance Ca^2+^‐dependent CaMKII, thereby regulating the transcription factor NFAT (nuclear factor that activates T cells).^45^ In our study, miR‐26a‐5p downregulated the expression of intracellular CaMKII by decreasing Wnt5a expression, ultimately affecting the transcription factor NFAT and then inhibiting the noncanonical Wnt signaling pathway. Generally, Wnt5a regulates the phosphorylation of calcium/calmodulin‐dependent protein kinase II (CaMKII) by inducing Ca^2+^ flux.^76^ Additionally, previous research also suggested that the Wnt5a could directly decrease CaMKII of inflamed macrophages. Recent study found that disrupted Wnt5a could downregulate the CaMKII and phosphorylated CaMKII, which indicated that the decreased phosphorylated CaMKII may at least partly result from the decreased CaMKII protein level.^77^ In the current study, we adopted a similar LPS‐induced myeloid cell lines to mimic the inflammatory situation as Pereia et. al^44^ and then we also found that a deceased whole protein level of CaMKII is correlated with the pro‐inflammatory cytokines. NFAT is initially present in nuclear extracts of activated T cells and is expressed in a variety of cells.^78^, ^79^ In the nervous system, NFAT is expressed in neurons and glial cells. Neuronal NFAT isoforms are involved in the regulation of neuronal survival, apoptosis, and axonal outgrowth during development or injury.^80^ NFAT is also expressed in primary microglia and leads to a pro‐inflammatory response.^79^, ^81^ Previous studies have shown that loss of NFAT alters the expression of several genes, including Itgam, Tnf, Il‐1b, and c‐Myc. NFAT directly mediates the upregulation of TNF‐α and IL‐1β.^82^ Our study also found that after the noncanonical Wnt signaling pathway was inhibited, the levels of IL‐1β, IL‐6, and TNF‐α in the spinal cord decreased and at the same time attenuated the neuroinflammation induced by microglia. Furthermore, in the current study, we focused on the status and function of microglia during the inflammatory pain, but neurons also may involve in the analgesia of miR‐26a‐5p by targeting Wnt5a, which is shown in Figure 3E and still needs further study to investigate the related mechanism of Wnt5a in neurons and neuron–microglia interaction under inflammatory pain background. Taken together, Wnt5a and the noncanonical Wnt signaling pathway act as a vital role in inflammatory pain, which is a promising target for developing miR‐26a‐5p‐related biomedicine.

In the current study, we also explored the administration route of miRNA. Our results showed that intrathecal injection has better antiinflammatory and biological effects than local subcutaneous administration. However, intrathecal drug delivery is often associated with complications such as bleeding, infection, and even death in severe cases.^47^ Subcutaneous administration in the hind paw is less invasive and has fewer side effects for administration than intrathecal injection. When options are available, patients tend to opt for a less invasive approach to treatment. Therefore, how to improve the effectiveness of subcutaneous administration is a direction for our future research. We can see that subcutaneous administration of miR‐26a‐5p agomir in the hind paw can also inhibit the activation of microglia in the dorsal horn of the spinal cord, and intrathecal injection of miR‐26a‐5p agomir can also reduce the subcutaneous inflammation in the hind paw. The way in which the mutual regulation of central‐peripheral inflammation is carried out is also worthy of our further study.

## 5 CONCLUSIONS

In conclusion, we reported that miR‐26a‐5p is a promising candidate for CFA‐induced inflammatory pain. We also compared the analgesic effect of intrathecal and subcutaneous injection of miR‐26a‐5p for inflammatory pain. Both administration methods could reduce the subcutaneous tissue and spinal cord inflammation. However, intrathecal injection produces longer analgesic period than local administration. Therefore, it still needs further research to develop an optimal administrate regimen for local injection, which may facilitate to avoid the adverse events result from intrathecal injection. Moreover, after intrathecal injection, miR‐26a‐5p regulated Wnt5a/CaMKII/NFAT, the noncanonical Wnt signaling pathway, of microglia in ipsilateral spinal cord to produce analgesia through antineuroinflammation (Figure 7).

## AUTHOR CONTRIBUTIONS

ZQ, JT and TT designed the study. YL, ML, and XG performed the experiments. PW and FZ helped implement Western blot and RT‐qPCR experiments. HW provided the venue for behavioral research and assisted in the completion of the von Frey test. YL drew the manuscript. YL, XG, and TT revised the paper. All authors have read and agree to the published version of the manuscript.

## FUNDING INFORMATION

This research was funded by the National Natural Science Foundation of China (Grant Number: 81973305), and Science and Technology Planning Project of Guangzhou, China (Grant Number: 201904010487), Natural Science Foundation of Guangdong Province, China (Grant Number: 2021A1515010897), and Discipline Construction Fund of Central People's Hospital of Zhanjiang (2020A01 and 2020A02).

## CONFLICT OF INTEREST STATEMENT

The authors declare that they have no competing interests.

## Supporting information


Appendix S1

## Data Availability

The data and materials supporting the conclusions of this study are available from the corresponding author on reasonable request.
