# Supplementary figures and images for: miR‐26a‐5p alleviates CFA‐induced chronic inflammatory hyperalgesia through Wnt5a/CaMKII/NFAT signaling in mice
Source: CNS Neurosci Ther. 2023 Feb 8;29(5):1254–71. doi: 10.1111/cns.14099 (PMC10068476; doi:10.1111/cns.14099)

Figure 4A

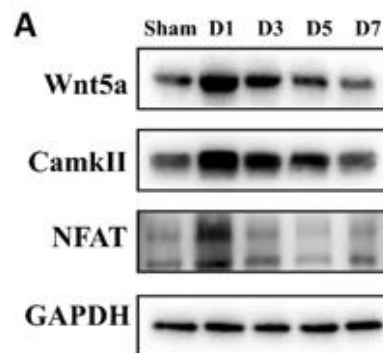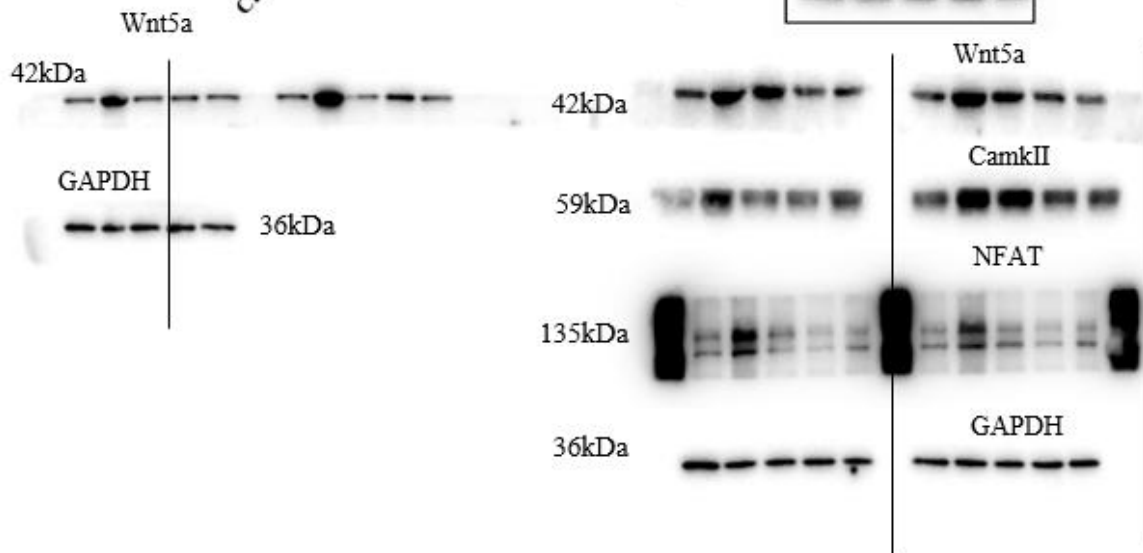

Figure 4F

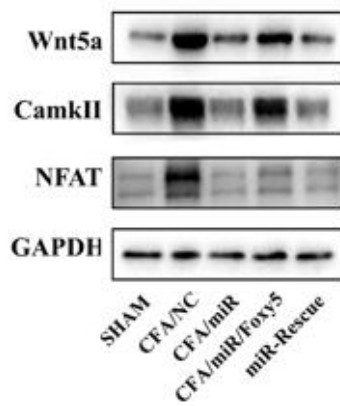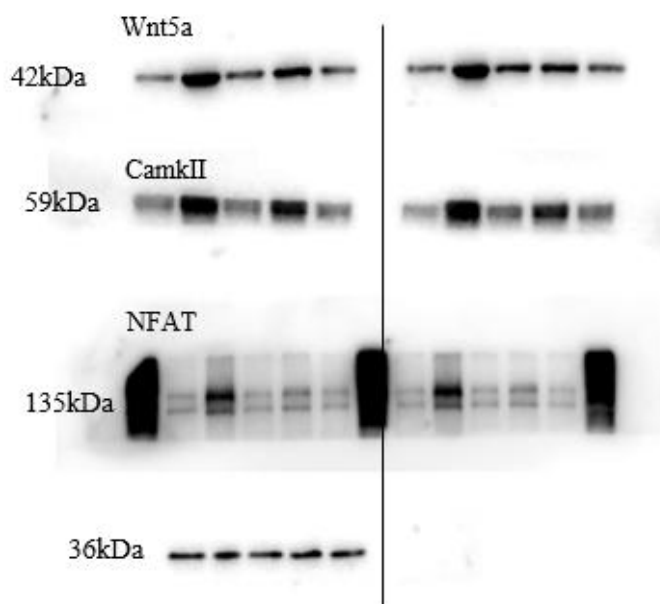

Figure 5B

**B**

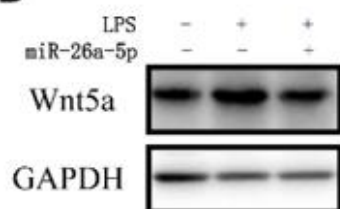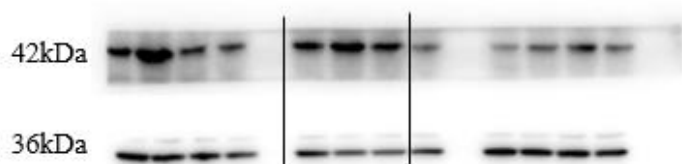

Figure 5F

**F**

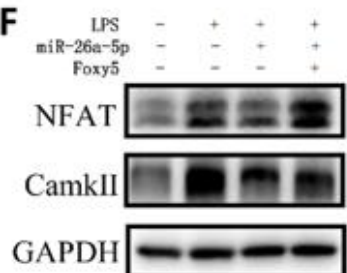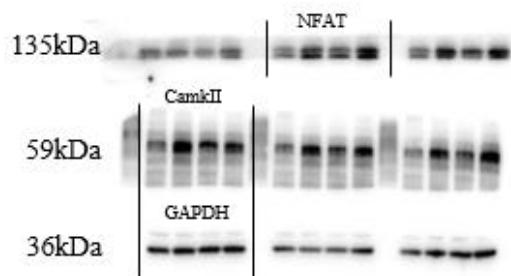

Figure 5G

**G**

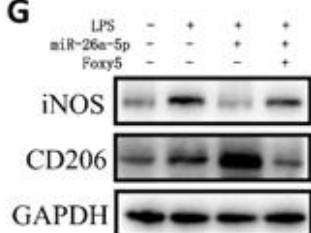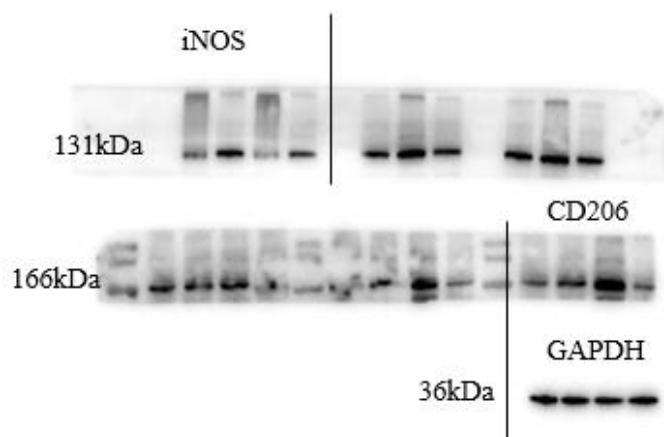

Supplement: Supplementary file 1 — Appendix S1 [file CNS-29-1254-s001.pdf]
